# Editorial: Tumor metabolism and programmed cell death

**DOI:** 10.3389/fendo.2024.1368737

**Published:** 2024-01-25

**Authors:** Dan-Lan Pu, Qi-Nan Wu

**Affiliations:** ^1^ Endocrinology Department, The People’s Hospital of Yubei District of Chongqing City, Chongqing, China; ^2^ Endocrinology Department, Dazu Hospital of Chongqing Medical University, The People’s Hospital of Dazu, Chongqing, China

**Keywords:** tumor, metabolism, programmed cell death, immunies, endocrinology

In recent years, extensive progress has been made in tumor endocrine and metabolism research, leading to the development of many translational medicine achievements, such as the identification of causal relationships between hormone-, growth factor-, glucose- and lipid metabolism-related signaling pathways and tumor occurrence and development; the identification of various endocrine signaling markers and tumors; the development of agents with specific targets, such as tumor cell receptors, kinases, and molecular structures; the development of therapeutic drugs that directly or indirectly block signaling pathways to regulate the cell cycle and life; the identification of endocrine- and metabolic-related adverse reactions induced by tumor-related treatments; the identification of abnormal nutritional metabolism; and the development of palliative support options for cancer patients.

Abnormal hormone secretion and energy metabolism may play important roles in the multiple programmed death processes of tumor cells and are closely related to the occurrence, development, metastasis, and treatment of tumors; moreover, tumor treatment may affect the endocrine metabolic system and programmed cell death, which leads to a poor prognosis (1). However, the underlying mechanism linking tumor metabolism and programmed cell death and the significance of this link have not been fully elucidated.

In 2019, Malireddi named a novel programmed cell death pathway, PANoptosis, an inflammatory programmed cell death pathway activated by specific triggers and regulated by the PANoptosome complex, which integrates key features of cell pyroptosis, apoptosis, and/or necroptosis (2). However, the role of PANoptosis in the field of breast cancer has not been explored. For this purpose, He et al. downloaded breast cancer data from The Cancer Genome Atlas (TCGA) databases and a single-cell sequencing dataset from the Gene Expression Omnibus (GEO) (GSE176078) to obtain PANoptosis-associated genes. COX and LASSO regression were used to identify PANoptosis-associated genes with prognostic value, and immune infiltration analysis and differential enrichment analysis of biological functions were performed. These studies indicated the potential of PANoptosis-based molecular clustering and prognostic features for predicting the prognosis of breast cancer patients. These findings could lead to new directions for immunotherapy in breast cancer patients.

Guo et al. searched for literature on lymphoma metabolism-related studies published in the last decade using scientific econometric indicators and information visualization techniques. The authors analyzed the temporal and spatial distribution of these studies, as well as the authors, journals, countries, affiliated institutions, their evolution, and network connectivity. These authors revealed a wealth of potential targets and drugs for treating lymphoma that merit further investigation for eventual translation into the clinic. These findings suggested that PI3K/AKT/mTOR and Bcl2 are important markers of metabolic abnormalities in lymphoma, while *Helicobacter pylori* is weakly associated only with gastric mucosa-associated lymphoid tissue lymphoma. Interestingly, the findings of the study indicated that “lymphoma, PET/CT, prognosis”, “cell apoptosis, metabolism, chemotherapy”, and “genotoxicity, mutagenicity” are crucial metabolic markers for lymphoma, but further exploration is needed to determine the potential of these research markers in the field of lymphoma metabolism. This study provided a comprehensive overview of the evolution of global research on lymphoma metabolism and the links among studies. Several significant research signatures related to lymphoma metabolism have been identified, and several underexplored areas that are essential for lymphoma metabolism still need further exploration. The scientometrics and visualization study method-Walktrap, an algorithm based on the random walk strategy, served as a robust tool for this study.

Previous studies revealed that metabolic reprogramming may be the key feature of clear cell renal cell carcinoma (ccRCC) and that altered gene expression may lead to significant metabolic changes that fuel tumor growth. Zhu et al. reviewed recent advances in understanding metabolic changes in ccRCC, including glucose, lipid, mitochondrial, and amino acid metabolism (glutamine, tryptophan, arginine). Some potential metabolic drug targets, such as HIF-2a inhibitors, fatty acid synthase (FAS) inhibitors, glutaminase (GLS) inhibitors, indoleamine 2,3-dioxygenase (IDO) inhibitors, and arginine depletion agents, are highlighted, and future trends in drug development are proposed; these trends include the use of combination therapies and personalized medicine approaches. The author indicated that ccRCC is characterized by significant metabolic reprogramming, which leads to changes in energy demand and redox homeostasis. During this process, cancer cells mainly utilize anaerobic glycolysis and HIF-driven lactate metabolism to inhibit the tricarboxylic acid cycle. A high lactate concentration in the tumor microenvironment promotes immune suppression and migration, while tryptophan breakdown leads to an increase in the synthesis of immunosuppressive kynurenine metabolites. The synthesis and utilization of lipids increase in ccRCC tissues vs. normal tissue, while lipid oxidation is inhibited. However, glutamine absorption increases to promote the production of fatty acids and counteract oxidative stress and ROS. Therefore, additional research is needed to reveal the mechanism of metabolic changes in ccRCC and develop targeted treatment strategies. At present, there are limited therapeutic options in clinical practice, and seeking to selectively impair tumor cell proliferation by targeting tumor-specific metabolic pathways could be a feasible strategy for developing new therapies for ccRCC. The efficacy and selectivity of these antimetabolic drugs are linked to the mutation profile of tumor cells, which alters cellular metabolism and induces cellular proliferation. Although the benefits of developing antitumor cell metabolism drugs outweigh the drawbacks of traditional antiangiogenic drugs, these agents may be safer for treating ccRCC and may reduce the incidence of cardiovascular side effects. In addition, metabolomics may aid in the development of targeted treatment methods for ccRCC. With the emergence of new metabolic technologies, authors anticipate an increase in the number of treatment options for chronic renal cell carcinoma soon.

Immune checkpoint inhibitor-induced isolated adrenocorticotropic hormone (ACTH) deficiency (IAD) is a rare but potentially fatal disease. Wang et al. searched PubMed and performed a systematic review of immune checkpoint inhibitor-induced isolated adrenocorticotropic hormone deficiency. The results indicated that most cases of IAD are related to the use of anti-PD-1 and anti-PD-L1 antibodies and should be differentiated from immune checkpoint inhibitor related pituitary dysfunction. The importance of prompt diagnosis and treatment of IAD should be fully recognized.

This Research Topic received a total of 16 submitted manuscripts, and 4 of those were published after a strict peer review process. We hope our readers will benefit from this work.

## Author contributions

D-LP: Conceptualization, Funding acquisition, Writing – original draft. Q-NW: Conceptualization, Formal analysis, Funding acquisition, Supervision, Writing – original draft, Writing – review & editing.
